# Improvement of a Cohesive Zone Model for Fatigue Delamination Rate Simulation

**DOI:** 10.3390/ma12010181

**Published:** 2019-01-07

**Authors:** Alessandro Pirondi, Fabrizio Moroni

**Affiliations:** Dipartimento di Ingegneria e Architettura, Università di Parma, Parco Area delle Scienze 181/A, 43124 Parma, Italy

**Keywords:** cohesive zone modelling, fatigue crack growth, finite element analysis, bonded interfaces

## Abstract

The cohesive zone model (CZM) has found wide acceptance as a tool for the simulation of delamination in composites and debonding in bonded joints and various implementations of the cohesive zone model dedicated to fatigue problems have been proposed in the past decade. In previous works, the authors have developed a model based on cohesive zone to simulate the propagation of fatigue defects where damage acts on cohesive stiffness, with an initial (undamaged) stiffness representative of that of the entire thickness of an adhesive layer. In the case of a stiffness that is order of magnitude higher than the previous one (for instance, in the simulation of the ply-to-ply interface in composites), the model prediction becomes inaccurate. In this work, a new formulation of the model that overcomes this limitation is developed. Finite element simulations have been conducted on a mode I, constant bending (constant G)-loaded double cantilever beam (DCB) joint to assess the response of the new model with respect to the original one for varying initial stiffness K_0_ and cohesive strength σ_0_. The results showed that the modified model is robust with respect to changes of two orders of magnitude in initial stiffness and of a factor of two in σ_0_.

## 1. Introduction

Composite and hybrid metal/composite structures are nowadays present not only in the aerospace industry, but thanks to continuous performance improvement and cost reduction, also many more industrial fields are approaching the use of multimaterial structural elements. This requires, in turn, extensive use of adhesive bonding and a more sophisticated capability to simulate and predict the strength of bonded connections where, for this purpose, analytical methods are being progressively integrated or replaced by finite element analysis (FEA). In engineering applications, it is well established that fatigue is the root cause of many structural failures. In the case of bonded joints, fatigue life is related to the initiation and propagation of defects starting at the free edges of joining regions or other features, such as through-thickness holes. In the case of composite or metal/composite joints, fatigue can start also from defects at the same locations cited above, with the difference that the crack may either run into the adhesive or become a delamination crack. In particular in the case of damage tolerant or fail safe design, it is necessary to know how cracks, or defects in general, propagate during the service life of a component.

**Figure 1:**
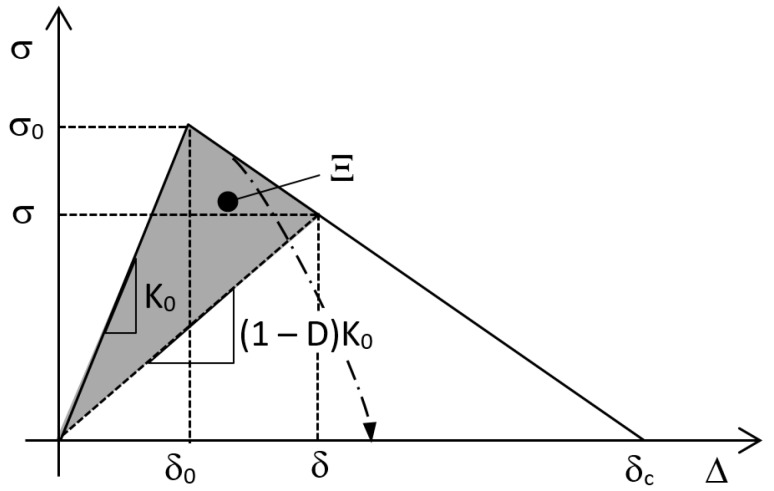
Example of bi-linear quasi-static (solid line) and subcritical, fatigue cohesive stress evolution (dashed-dotted line).

Originally proposed by Barenblatt [1], the cohesive zone model is extensively used for the prediction of fracture propagation under quasi-static conditions along the interfaces. Considering a bi-linear cohesive law (see for example Figure 1) in a quasi-static simulation, the interface behaves linearly up to δ_0_. Above this value, the stiffness of the interface is progressively reduced in order to yield the descending branch of the law until the critical opening δ_c_ is attained. In this phase, the unloading follows the dashed line. The cohesive zone model was further developed in a discrete damage zone model and integrated with the extended finite element method (XFEM) by Wang et al. [2] in order to simulate the crack propagation in an arbitrary direction.

In the literature, a certain number of works can be found dealing with the simulation of the fatigue crack propagation using the cohesive zone model, many of them being recently reviewed in [3,4]. When dealing with fatigue, crack growth may occur also sub-critically, that is at a value of stress lower than that shown by the solid line in Figure 1. Among cyclic cohesive models, some were applied to the modelling and prediction of fatigue in bulk, ductile materials [5,6,7,8,9,10,11,12,13,14,15,16] or quasi-brittle polymers [17] while, at the same time, several others were developed with reference to applications involving failure at the interfaces, such as debonding in adhesive joints or delamination and matrix cracking (followed by delamination) in polymer composites [18,19,20,21,22,23,24,25,26,27,28,29,30,31,32,33,34,35,36,37].

When modelling fatigue, an important point is the integration of the damage (hence crack growth) rate. Most of the models for bulk, ductile or quasi-brittle materials [6,7,8,9,10,11,12,13,14,15,16,17] as well as some for interfaces [18] rely on a full incremental solution of each individual load cycle, eventually adopting some kind of extrapolation scheme in order to reduce the computation time (see for example [8]). Others adopt instead a load envelope strategy (simulation is performed applying the maximum load of the cycle without any unloading-reloading) [19,20,21,22,23,24,25,26,27,28,29,30,31,32,33,34,35,36,37] to save computation time. In these types of model, damage evolution equations are formulated in terms of damage rate per load cycle and they generally include external load parameters (like load ratio) in the damage evolution equation. They are, therefore, defined as non-constitutive [38] to differentiate them from models ([5,6,7,8,9,10,11,12,13,14,15,16,17] and others reported in [38]) where a constitutive traction-displacement behavior was formulated with evolution equations for the internal variables accounting for the possibility of cyclic damage accumulation. The model developed by the authors in [27,31,36] belongs to the non-constitutive kind, and therefore the analysis of the literature will be restricted in the following to the other models of this kind.

The cohesive zone damage evolution under fatigue loading is accounted for in different ways. One first way is to establish a priori a phenomenological law [19,20,22,23,24] where fatigue damage, D_f_, sums up with the quasi-static damage, D_s_, defined as,

(1) $$D_{s}=\frac{\delta_{c}}{\delta }\frac{\delta -\delta_{0}}{\delta_{c}-\delta_{0}}$$

to yield the overall effect of fatigue loading to the cohesive zone. In this case, the fatigue damage evolution law contains parameters that have to be adjusted to calibrate the numerical model with experimental results, usually by trial and error, with possible limitations on the simulation of different conditions.

A different approach to fatigue damage was formulated in [21], where a link between damage and fracture mechanics was established assuming that the crack growth rate dA/dN is equal to the sum of the damaged area growth rates of i-th elements in the cohesive zone, i.e.,

(2) $$\frac{\mathrm{dA}}{\mathrm{dN}}=\underset{A_{\mathrm{CZ}}}{\sum }\frac{{\mathrm{dA}}_{d}{}^{i}}{\mathrm{dN}}$$

coming to the following relationship for cyclic damage:

(3) $$\frac{\mathrm{dD}}{\mathrm{dN}}=\frac{1}{A_{\mathrm{CZ}}}\frac{{\left[\left(1-D\right)\delta_{c}+{D\delta }_{0}\right]}^{2}}{\delta_{0}\delta_{c}}\frac{\mathrm{dA}}{\mathrm{dN}}$$

while the quasi-static component of damage is still given by Equation (1) and the overall damage rate is of course the sum of the quasi-static and fatigue rates. In this way, the fatigue damage rate can be determined directly from the experimental fatigue delamination rate dA/dN. The last, in fact, can be expressed as a function of the cyclic strain energy release rate, ∆G, by means of a Paris-like equation

(4) $$\frac{\mathrm{dA}}{\mathrm{dN}}=B\Delta G^{d}$$

where ΔG = (1 − R^2^)G_max_ and G_max_ is maximum value of G of the cycle. However, in this model the damage variable D representing the loss of stiffness, i.e., $K=\left(1-D\right)K_{0}$, does not coincide with the density of microcracks on a representative interface element, $D=A_{d}/A_{e}$, as it may be deduced from the application of the effective stress concept and strain equivalence principle of continuum damage mechanics (see [39]). Rather, the density of microcracks on a representative interface element is related to the damage variable representing the loss of cohesive strength $\sigma =\left(1-\bar{D}\right)\sigma_{0}$ (linear softening). This implies also that $\bar{D}$ is coincident with the ratio of the energy dissipated during the damage process, Ξ, to the critical energy release rate, G_c_.

(5) $$\bar{D}=\frac{A_{d}}{A_{e}}=\frac{\Xi }{G_{c}}=1-\left(1-D\right)\frac{\delta }{\delta_{0}}$$

The same kind of approach was adopted in [25,26,28,29,30,32], although coming to a slightly different formulation of the fatigue damage rate:

(6) $$\frac{\partial D_{f}}{\partial N}=\frac{1-D_{s}-D_{f,u}}{A_{\mathrm{fat}}}\frac{\mathrm{dA}}{\mathrm{dN}}$$

in 25, 26, 28 and 29, where D_f,u_ accounts for unwanted fatigue damage, i.e., fatigue damage that occur before the value of δ in Figure 1, and,

(7) $$\frac{\partial D_{f}}{\partial N}=\frac{1-D_{s}}{A_{\mathrm{eff}}}\frac{\mathrm{dA}}{\mathrm{dN}}$$

in [32], while [30] exploited Equation (3). In these works, an effort was also put on the development of alternative ways to identify the values of A_CZ_ (A_eff_) and ∆G with respect to [21], where A_CZ_ was estimated using mode I Rice’s closed-form equation and simulations were performed for geometries where ∆G was independent of crack length. In particular, [32] defined A_eff_ as the portion of the area on the crack tip element subjected to fatigue damage, while [30] adopted a modified form of Rice’s solution that accounts for the difference in A_cz_ between mode I and mode II loading. In both works, the simulation was done on geometries where G was dependent on the crack length.

In [25,26,28,29], A_CZ_ was evaluated numerically by performing a quasi-static analysis before going on to the fatigue damage step. The quasi-static cohesive zone length, A_CZ,s_ was extracted as the sum of the areas of elements where 0 < D < 1 and, since fatigue crack growth (FCG) occurs at a value G < Gc, A_CZ_ was finally estimated as:

(8) $$A_{\mathrm{CZ}}=\left(\frac{G}{G_{c}}\right)A_{\mathrm{CZ},s}$$

Concerning the calculation of the fatigue damage rate, A_fat_ in Equation (6) is the portion of A_CZ_ where damage develops subcritically, that is at a stress lower than the one dictated by the quasi-static traction-displacement behavior. The value of A_fat_ was taken equal to half of A_CZ_ based on the results of simulation examples. The value of A_eff_ in Equation (7) corresponds instead to the undamaged length of the crack tip element when a new loading is applied to the model. For example, if D_s_ = 0.4 at the crack tip cohesive element after a quasi-static loading, the element will enter the fatigue loading step with a 60% (A_eff_ = 0.6A_e_) of residual area. In [30], a closed-form formulation of A_CZ_ was adopted alike [21] but depending in this case on the mode mixity.

Regarding ∆G, the authors of [25,26,28,29,30] used the instantaneous (i.e., at the current increment) integrated traction-displacement response of cohesive elements, where the subcritical portion (see Figure 1) was assumed to be vertical. It is worth underlining that these models calculate ∆G pointwise within A_CZ_, and therefore the fatigue damage rate varies pointwise accordingly. In other words, the experimental fatigue crack growth rate dA/dN evaluated at the scale of the specimen is used to model local damage processes. The authors of [32] choose to concentrate fatigue damage into the crack tip element; therefore, the value of G was evaluated in the same way as in [25,26,28,29,30] but only at the crack tip element. Since in this way G increased with delamination growth contradicting experimental results, the value considered was calculated as a weighted average of the values of G at the crack tip and at the element just ahead of the crack tip. In [33] the authors recognized that the “instantaneous” G may vary with fatigue degradation and mesh refinement, while the value of G at the cohesive element failure is a better estimate. The strain energy release rate at failure was, therefore, extracted from elements in the wake of the numerical crack front, assuming that the variation of G between consecutive cohesive elements is small.

All of the models mentioned above consider the development of damage starting from the softening branch of the cohesive law. Since fatigue damage is likely to begin below the quasi-static stress threshold σ_0_, or it is possible that it develops in absence of a pre-existing crack (i.e., cohesive element would be little stressed in this case), a condition for crack initiation was added in [26]. Here a fatigue damage initiation parameter d_fi_ was accumulated, depending on the stress level of cohesive elements with respect to fatigue stress-life behaviour. In order to identify correctly the initiation region, a failure index was also introduced [28] and assessed [29].

The definition of damage of Equation (5) and the link between damage mechanics and fracture mechanics represented by Equation (2) were adopted also in [34,35]. They however defined an equivalent δ that incorporates the cumulative effects of the static (D_s_) and fatigue (D_f_) damage on the material stiffness, as the variable to be continuously recorded during the analysis. In this way the damage, independently of its origin, follows Equation (1) where D_s_ is replaced by D. The resulting fatigue damage evolution is:

(9) $$\frac{\partial D_{f}}{\partial N}=\frac{r_{w}}{A_{\mathrm{CZ}}}\frac{\delta_{0}\delta_{c}}{\delta^{2}}\frac{\mathrm{dA}}{\mathrm{dN}}$$

where r_w_ represents the relative weight of each Integration Point (IP) in the Newton–Cotes integration scheme chosen by the authors. The three IPs of each cohesive element used here, have therefore relative weights of (1, 4, 1), respectively. With a few algebraic manipulations, it can be shown that Equation (9) is equivalent to Equation (5) except the presence of the weight r_w_. Again, G is evaluated at each point within A_CZ_ implying that the macroscopic fatigue crack growth behavior still holds at the mesoscale.

Also the model developed in [27,31,36] made use of the same link between damage and fracture mechanics established in [21], i.e., Equation (2). As in other works cited before, a focus was done on the improvement of the precision of the model, namely [27]:

- evaluation of G as a whole model value using the contour integral;
- numerical evaluation of A_CZ_ increment-by-increment during the simulation;
- introduction of a threshold for fatigue crack growth, δ_th_, that can be lower than the quasi-static stress threshold δ_0_.

**Figure 2:**
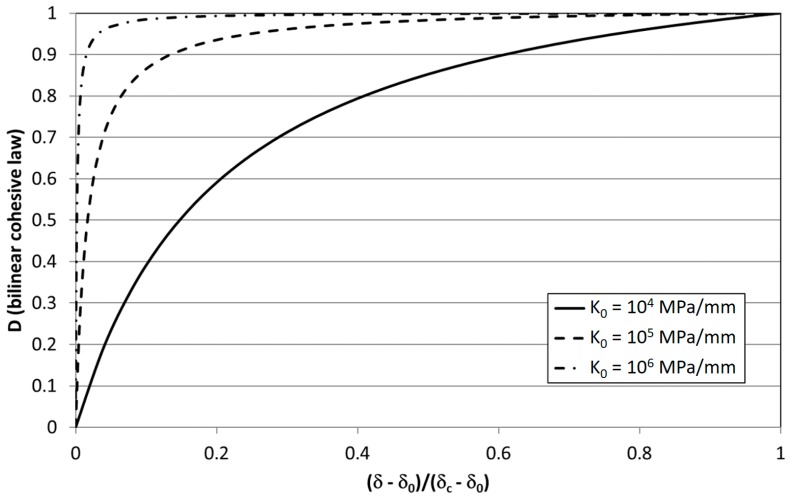
Evolution of quasi-static damage, Equation (1), for different values of K_0_ and σ_0_ = 30 MPa.

Mixed-mode I/II criteria were then implemented in [31] and three-dimensional crack fronts were managed in [36]. The effects of the density of microcracks on a representative interface element, A_d_/A_e_ was related to the loss of stiffness, i.e., D = 1 − K/K_0_, and the fatigue damage evolution law was developed on this basis resulting:

(10) $$\frac{{\mathrm{dD}}_{f}}{\mathrm{dN}}=\frac{1}{A_{\mathrm{CZ}}}\frac{\mathrm{dA}}{\mathrm{dN}}=\frac{1}{A_{\mathrm{CZ}}}B\Delta G^{d}$$

while quasi-static damage follows Equation (1). Stiffness-based damage is one of the damage measures found in the literature; other measures for definition of damage include [38]: (i) current traction; (ii) current separation; (iii) energy dissipated during the monotonic loading process up to the current state. All of these types were found in the models analysed previously (stiffness was used also in [19,20]). In [38] it has been illustrated, for the case of the bilinear cohesive law, that these damage measures can be uniquely converted one another. Among the different measures, stiffness-based damage is quite sensitive to the value of initial stiffness, i.e., the higher K_0_, the steeper the damage evolution. In [40] a study was performed in order to define bound values of the initial stiffness for a cohesive zone model. The sensitivity of the damage evolution with respect to the initial stiffness is quite clear looking at the graphical representation of Equation (1) in Figure 2 for increasing values of K_0_. This implies that, using this measure, a high value of damage may not necessarily mean a high loss of strength. In [38] it was also pointed out that an energetic damage variable may be an appropriate quantity to interpret the damage states but the formulation of cohesive laws with this measure is not recommended. Instead, the use of the separation-type damage variable is more effective and efficient for this purpose.

The development of the model [27] was done focusing on bonded joints. In finite element modelling of bonded joints, the adhesive layer, although not infinitely thin, is generally not introduced explicitly in the finite element model because of modelling and computation time convenience. The adhesive layer behavior and damage is therefore embedded into the cohesive zone, that has the purpose not only to model decohesion but also the stiffness of the bondline which may not be as high as usually assumed in CZM. For instance, a typical figure would be a 0.2 mm thick layer of an epoxy adhesive having an elastic Young modulus equal to 2000 MPa, that yields a stiffness K_0_ = 2000/0.2 = 10^4^ MPa/mm. This value is 2–3 order of magnitude lower than that used typically to model nominally zero-thickness interfaces with CZM. Looking at Figure 2, damage evolution with such a value of K_0_ may not be drastically steep as with order of magnitudes higher values. A stiffness-based damage variable is also used in the commercial finite element program ABAQUS, where the model [27,31,36] was implemented by means of the embedded user subroutines USDFLD and URDFIL. These motivations can justify from the numerical side the use of a stiffness-based damage measure.

Damage in the adhesive layer can develop in the form of microcavities and crazes as a result of the presence of filler particles and of the nature of the polymer matrix. Therefore, a fracture occurs in several cases in a quasi-brittle fashion. For this reason, it is believed that the use of a stiffness-based damage can be justified in this case also from a physical point of view. This might not be the same for very thin (ideally infinitely thin) interfaces such as metal-ceramic joints or, depending on the retained resin content after curing, ply-to-ply interface in composite laminates. In those cases, very high values of stiffness can be expected, that can be hardly evaluated in practice; therefore, working with a definition of damage based on the loss of interface (cohesive) strength might be more appropriate from the physical standpoint.

Recently, Reference [41] compared the performance of six models [19,21,25,31,33,37]. The conclusions drawn by this benchmark study are that all the models, with a few exceptions [37,33] are influenced by parameters not directly linked to the damage rate model, which significantly reduce their capabilities to perform effective simulations on a broad range of parameters. In particular concerning [31], the dependence of simulation results on the initial stiffness K_0_ and on the cohesive strength σ_0_ were indicated as the main responsible of the inaccuracy. It is worth underlining that in [31] and related papers, the initial stiffness was always set to K_0_ = 10^4^ MPa/mm, as the target of the application was on adhesive joints. When the authors of [41] used this value in the simulations with the model of [31], they found a good agreement between theoretical and simulated FCG rate.

The objectives of this work is therefore to develop and validate a new formulation of the model [27] that would not be sensitive to changes in K_0_ and σ_0_ and, therefore, demonstrate that a stiffness-based damage measure is suitable also in the case of stiff interfaces. The steps are:

- to identify the root causes of the poor performance of the model at high values of K_0_;
- to review critically the implementation strategy;
- to propose a revised version of the model;
- to assess the response of the revised model for varying K_0_ and σ_0_.

## 2. Model Implementation into Abaqus™

The model [27] was implemented into the software Abaqus™ 6.13 (Dassault Systèmes, Vélizy-Villacoublay, France) by programming the user subroutines USDFLD and URDFIL. The USDFLD subroutine allows to define a field-variable dependent material behaviour, such as the dependence of stiffness on damage, while URDFIL is used to post-process results during the analysis, in this case to obtain G from stress and opening of cohesive elements and to update the number of elapsed cycles. A field variable dependence through USDFLD can be easier to implement than a user-defined material behaviour by the UMAT subroutine, although at the expense of the introduction of an explicit solution dependence since USDFLD provides access to material point quantities only at the beginning of the increment. Therefore, the accuracy of the results may depend on the increment size, that is in this case on the value of ∆D_max_ set by the user.

**Figure 3:**
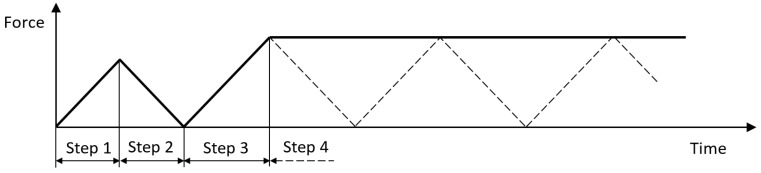
Outline of analysis steps.

The analysis is divided in four steps (see Figure 3):

1. ramp-up until $G_{\mathrm{th}}=\frac{\Delta G_{\mathrm{th}}}{1-R^{2}}$ and evaluate the fatigue damage threshold δ_th_ as the value of δ at the crack tip cohesive element;
2. ramp down to zero force and remove damage possibly developed in the previous step;
3. ramp up to the maximum load of the cycle;
4. simulation of fatigue phase in subsequent increments (j-th), with the procedure described in the following, while keeping the load constant along the increments; ΔG^j^ is evaluated using the contour integral over a path surrounding the cohesive zone. In this step, both static ad fatigue damages are considered.

**Figure 4:**
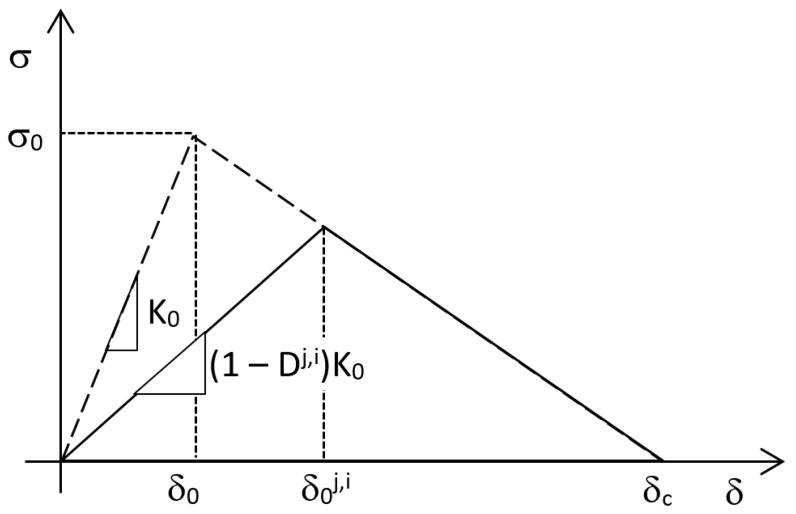
Cohesive law update.

The workflow of the algorithm used for the fatigue crack growth simulation during step 4 includes the following two phases, that are repeated sequentially every increment *j* of the analysis step:

1. Solution phase (USDFLD subroutine)
  For every integration point i of cohesive elements:
  Get σ^j,i^, δ^j,i^
  Initialize D^j,i^ = D^j−1,i^
  Update cohesive law: $\delta_{0}{}^{j,i}=\frac{\delta_{0}\delta_{c}}{\delta_{0}+\left(\delta_{c}-\delta_{0}\right)\left(1-D^{j,i}\right)}$ (see Figure 4)
  Update field variable FV = D^j,i^ and store it
  Loop over integration points of cohesive elements
2. Post-processing phase (URDFIL subroutine)
  Evaluate A_cz_^j^
  Evaluate G^j^ using contour integral; ∆G^j^ = G^j^(1 − R^2^)
  Initialize N^j^ = N^j−1^
  For every integration point i:
  Impose tentative damage increment $\Delta D^{j,i}=\min\left\{\Delta D_{\max};1-D^{j,i}\right\}$
  (∆D_max_ is a user-defined value)
  Calculate $\Delta N^{j,i}=\frac{\Delta D^{j,i}}{B{\left(\Delta G^{j}\right)}^{d}}A_{\mathrm{CZ}}{}^{j}$ Loop over integration points of cohesive elements
  For the entire model:
  Find $\Delta N^{j}{}_{\min}=\min_{i}\left\{\Delta N^{j,i}\right\}$
  Update damage N^j^ = N^j^ + $\Delta N^{j}{}_{\min}$ and store it for increment j + 1
  For every integration point i:
  Calculate $\Delta D^{j,i}=\Delta N^{j}{}_{\min}\frac{B{\left(\Delta G^{j}\right)}^{d}}{A_{\mathrm{CZ}}{}^{j}}$
  Update damage D^j,i^ = D^j−1,i^ + $\Delta D^{j,i}$ and store it for increment j + 1
  Loop over integration points of cohesive elements
  Damage is shared between subroutines in a Fortran COMMON block.

## 3. Model Performance Checkout

### 3.1. Modelling

**Figure 5:**
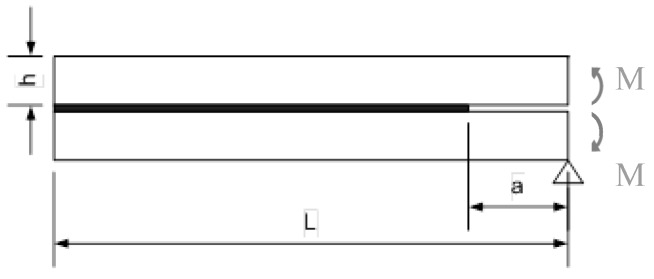
Joint geometry of simulations. Dimensions: a_0_ = 30mm; h = 5 mm; L = 335 mm; M = 300 Nmm/mm.

The fatigue crack growth rate dependence on model parameters is tested in the present work on a mode I double cantilever beam (DCB) joint geometry loaded with a constant bending moment, Figure 5, that yields a nominally constant G = M^2^/EI for increasing crack length (E is the adherends elastic modulus and I is the second moment of area of the beam section). The analysis is two-dimensional and only the upper cantilever was modelled due to symmetry. The cantilever is modelled with four-node, reduced integration plane stress elements, with an average mesh size of 1 mm (length direction) and 0.25 mm (thickness direction), respectively. The debonding interface instead, is represented through four-node cohesive elements. In order to mesh the process zone with enough accuracy (at least 4–5 elements enclosed in the cohesive process zone) even in the case of a very high cohesive stiffness, the cohesive element size has to be set to a low value, as well as it does for the maximum damage increment ∆D_max_. A preliminary study was done where four combinations of cohesive element size (0.2 or 0.02 mm) and ∆D_max_ (0.2 or 0.02) were tested, yielding that a cohesive element size of 0.02 mm and a ∆D_max_ = 0.02 is a reasonable trade-off to keep the accuracy of the solution as high as possible compatibly with the simulation time. A rigid kinematic coupling is set between the cantilever and the cohesive zone.

The stiffness of cohesive elements is dependent on the current value of damage according to the procedure described in Section 2. Cantilevers are assigned isotropic elastic constants E = 70 GPa and ν = 0.3. The CZM and FCG parameters that hold for all simulations are reported in Table 1. Fatigue is simulated with a load ratio R = 0 (i.e., ∆G = maximum G of the cycle).

The CZM parameters that are varied in the simulations are reported instead in Table 2. The values were chosen in the following way:

- K_0_ = 1 × 10^4^ MPa/mm is the initial stiffness adopted in all the works done previously by the authors, representing a 0.2 mm thick adhesive layer of a polymer with Young’s modulus equal to 2000 MPa. Other values were set one- and two-order of magnitude greater than that, in order to represent much more thin layer such ply-to-ply interface in composite laminates;
- load levels corresponding to G/G_c_ = 0.25, 0.5 and 0.75, respectively, were simulated for each of the values of K_0_;
- the influence of σ_0_ has been assessed only in the case of an intermediate value of initial stiffness to limit the number of simulations (see Table 2).

The introduction of δ_th_ instead of δ_0_ as a fatigue threshold has the effect of moving backward or forward the transition point between quasi-static and subcritical damage evolution, alike K_0_ and σ_0_. Since the extent of this effect depends, at the same time, on the values assumed by K_0_ and σ_0_, for the sake of simplicity a detailed analysis has not been carried out regarding δ_th_.

### 3.2. Results

**Figure 6:**
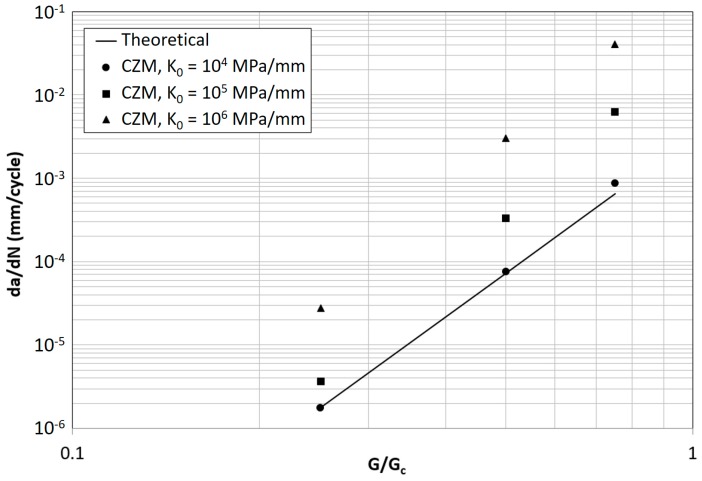
Theoretical and predicted FCG rate for different values of K_0_ and G/G_c_ (σ_0_ = 30 MPa).

The FCG rate predicted by the model for the parameter combinations in Table 2 is summarized in Figure 6. The simulated crack growth varied from about 1 mm to 5 mm depending on the FCG rate. A linear regression of crack length vs. number of cycles has been done in order to extrapolate the FCG rate and to evaluate the correlation of the simulation with the theoretical trend represented by Equation (4). At a value of K_0_ = 10^4^ MPa/mm the model yields results in line with the theoretical value, showing just a small deviation at the highest value of G/G_c_. For increasing values of K_0_, as shown in 41, an increasing deviation from the theoretical trend is found that may become very large. This confirms also why in previous works of the authors, where simulations were done with a low value of stiffness, representative of an adhesive layer (10^4^ MPa/mm), a good correspondence between theoretical and simulated FCG rate was always found.

**Figure 7:**
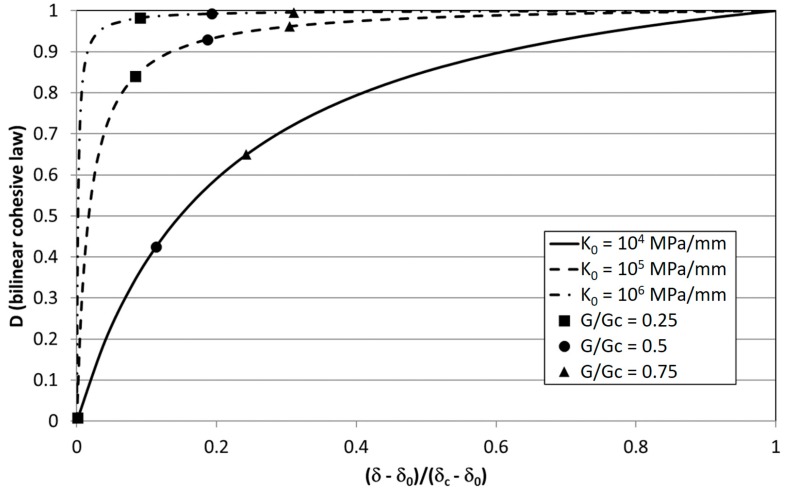
Evolution of quasi-static damage, Equation (1), for different values of K_0_ and σ_0_ = 30 MPa. Dots represent the transition point between quasi-static (“critical”) and subcritical fatigue damage evolution.

This behavior is consistent with the dependence of the transition point between quasi-static and subcritical fatigue damage with the value of K_0_ represented in Figure 7. The higher the initial stiffness and strain energy release rate, the higher is the amount of damage accumulated following the quasi-static cohesive law, i.e., non-subcritically.

**Figure 8:**
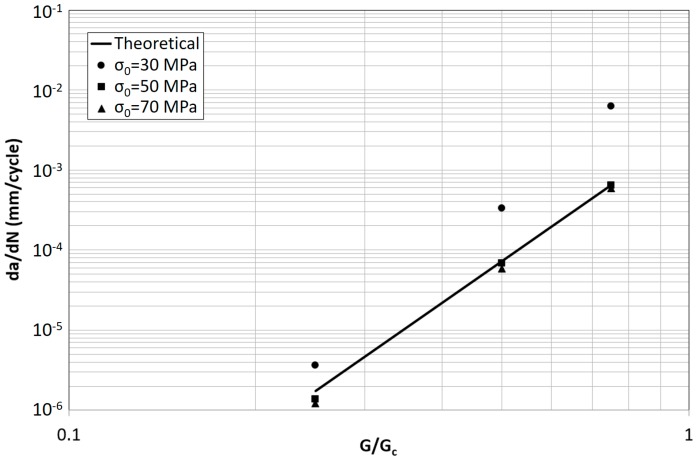
Theoretical and predicted FCG rate for different values of σ_0_ and G/G_c_ (K_0_ = 10^5^ MPa/mm).

Not surprisingly, the opposite trend is found in Figure 8 by increasing σ_0_, where the difference between simulation and theory decreases. Thus, the higher σ_0_ the lower the non-subcritical damage accumulation.

## 4. Modification of the Model and Validation

### 4.1. Modification

From the analysis conducted in Section 3, the model yields accurate predictions of the FCG rate under conditions of low values of K_0_ and G/G_c_ and high values of σ_0_, that is when damage accumulates mainly under subcritical conditions. Therefore, a term accounting for fatigue damage developing under “critical” (i.e., quasi-static) stress-opening conditions is foreseen.

**Figure 9:**
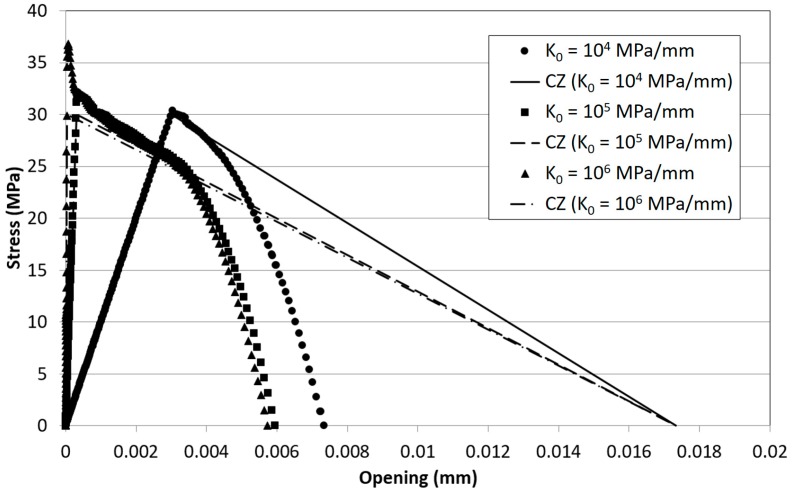
Stress as a function of opening in cohesive elements for G/G_c_ = 0.5.

The model [27] makes use of the Abaqus™ USDFLD subroutine for an easier implementation of a damage-dependent cohesive stiffness, with respect to the use of UMAT (User-defined material). However, as anticipated in Section 2, the cohesive element stiffness is not updated by USDFLD during the increment and the stress-opening distribution in the cohesive zone may therefore overshoot the quasi-static cohesive law. An example of this behavior is shown in Figure 9. The overshooting of the quasi-static limit corresponds to an unintended damage increment, ∆D*^j,i^, that is not accounted for in the procedure described in Section 2. Since the overshooting of the cohesive law tends to be more pronounced at high values of K_0_ and/or G/G_c_ a high discrepancy between theoretical and simulated FCG rate can be expected under these circumstances, as seen in Figure 6. A high stiffness leads also to a more pronounced overshooting of the cohesive law especially at the end of the elastic phase, always because of the explicit solution dependence introduced by the USDFLD subroutine.

The idea of a correction for unintended quasi-static damage was developed in [37], due in that case to the mesh-dependency of opening profile and traction field in the cohesive zone, and implemented by a novel predictor-corrector approach. The predictor step performed an approximate prediction of the total damage increment of ΔN and the corrector step handled the unintended evolution of quasi-static damage in terms of an adjustment of the number of loading cycles.

**Figure 10:**
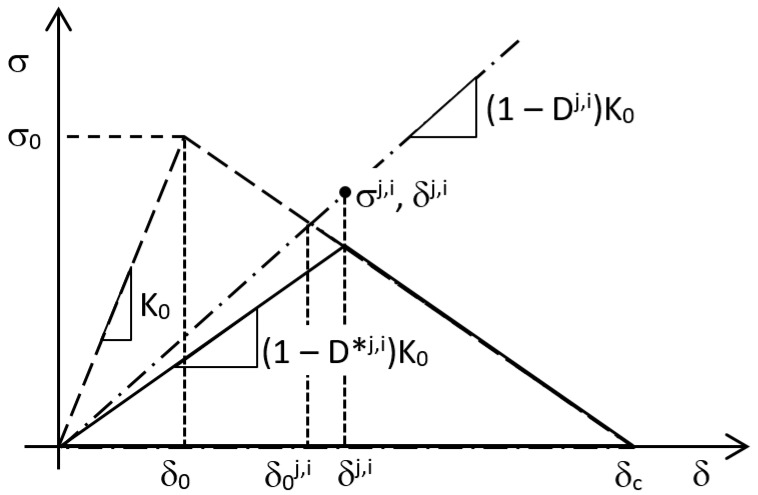
Outline of the definition of D*^j^.

For the sake of simplicity, a first-guess, direct estimate of the damage increment ∆D*^j,i^ is done referring to the scheme illustrated in Figure 10:

∆D*^j,i^ = D*^j,i^ − D^j,i^ (11)

where,

(12) $$D^{*j,i}=\frac{\delta_{f}\left(\delta^{j,i}-\delta_{0}\right)}{\delta^{j,i}\left(\delta_{c}-\delta_{0}\right)}\;\mathrm{if}\;\delta^{j,i}>\delta_{0}{}^{j,i}$$

(13) $$D^{*j,i}=D^{j,i}\;\mathrm{if}\;\delta^{j,i}\leq \delta_{0}{}^{j,i}$$

In other words, ∆D*^j,i^ represents an additional fatigue damage increment that is accumulated non-subcritically. The condition $\delta^{j,i}\leq \delta_{0}{}^{j,i}$ corresponds to subcritical fatigue crack growth where the stress lies below the cohesive law. The field variable where damage is stored for the following increment is then updated to the value of D*^j,i^.

Similarly to the corrector step in [37], the increment of damage ∆D*^j,i^ is then related to an increment of number of cycles ∆N*^j^. In this case, it has been done by defining an equivalent damaged area by the knowledge of $A_{e}{}_{i}$, the effective area of each of the i-th elements lying on A_CZ:_

(14) $$A_{d}{}^{*j}=\underset{i\in A_{\mathrm{CZ}}}{\sum }A_{e}{}_{i}\Delta D^{*j,i}$$

and dividing this latter by the fatigue crack growth rate, yielding,

(15) $$\Delta N^{*}{}^{j}={\left(\frac{A_{d}{}^{*}}{\mathrm{dA}/\mathrm{dN}}\right)}^{j}=\frac{\sum_{i\in A_{\mathrm{CZ}}}A_{e}{}_{i}\Delta D^{*j,i}}{B{\left(\Delta G^{j}\right)}^{d}}$$

that has to be added to ∆N^j^_min_ (see Section 2) to have ∆N^j^ = ∆N^j^_min_ + ∆N*^j^. The workflow is therefore:

1. Solution phase (USDFLD subroutine)
  For every integration point i of cohesive elements:
  Get σ^j,i^, δ^j,i^
  Initialize D^j,i^ = D^j-1,i^
  Update cohesive law: $\delta_{0}{}^{j,i}=\frac{\delta_{0}\delta_{c}}{\delta_{0}+\left(\delta_{c}-\delta_{0}\right)\left(1-D^{j,i}\right)}$
  Check for IPs exceeding quasi-static cohesive stress limit:
  if $\delta^{j,i}>\delta_{0}{}^{j,i}$ update D^j,i^ to D*^j,i^ = $\frac{\delta_{f}\left(\delta^{j,i}-\delta_{0}\right)}{\delta^{j,i}\left(\delta_{c}-\delta_{0}\right)}$
  Update field variable FV = D*^j,i^ and store it
  Loop over integration points of cohesive elements
2. Post-processing phase (URDFIL subroutine)
  Evaluate A_cz_^j^
  Evaluate G^j^ using contour integral; ∆G^j^ = G^j^(1 − R^2^)
  Initialize N^j^ = N^j-1^
  For every integration point i:
  Impose tentative damage increment $\Delta D^{j,i}=\min\left\{\Delta D_{\max};1-D^{*j,i}\right\}$
  Calculate $\Delta N^{j,i}=\frac{\Delta D^{j,i}}{B{\left(\Delta G^{j}\right)}^{d}}A_{\mathrm{CZ}}{}^{j}$
  Loop over integration points of cohesive elements
  For the entire model:
  Find $\Delta N^{j}{}_{\min}=\min_{i}\left\{\Delta N^{j,i}\right\}$
  For every integration point i:
  Calculate $\Delta D^{j,i}=\Delta N^{j}{}_{\min}\frac{B{\left(\Delta G^{j}\right)}^{d}}{A_{\mathrm{CZ}}{}^{j}}$
  Calculate ∆D*^j,i^ = D*^j,i^ − D^j,i^
  Calculate $\Delta N^{*}{}^{j}=\frac{\sum_{i\in A_{\mathrm{CZ}}}A_{e}{}_{i}\Delta D^{*j,i}}{B{\left(\Delta G^{j}\right)}^{d}}$
  Update damage D^j,i^ = D*^j,i^ + ∆D^j,i^ and store it for increment j + 1
  Loop over integration points of cohesive elements
  For the entire model:
  Update no. of cycles ∆N^j^ = ∆N^j^_min_ + ∆N*^j^ and store it for increment j + 1

The results of this modification will be assessed in the next section.

### 4.2. Results

**Figure 11:**
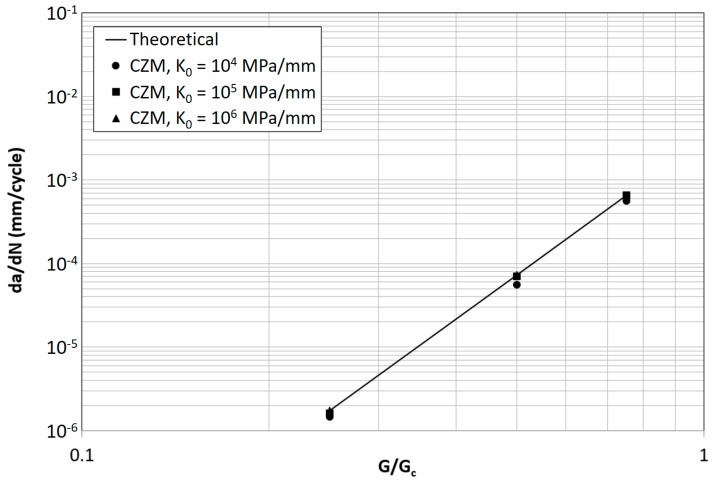
FCG rate as a function of G/G_c_ for different values of K_0_ (σ_0_ = 30 MPa).

The fatigue crack growth rate obtained by adding ∆N*^j^ to ∆N^j^_min_ is shown in Figure 11 as a function of G/G_c_. It is evident that now the FCG rate is coherent with the theoretical value (Equation (4)) independent of K_0_. Also the influence of G/G_c_ visible in Figure 6 is now absent. This is a confirmation that the dependence of the FCG rate on the value of initial stiffness in Figure 6 is related to the explicit solution dependence introduced by the use of the USDFLD subroutine, rather than on the choice of stiffness as a measure of damage.

**Figure 12:**
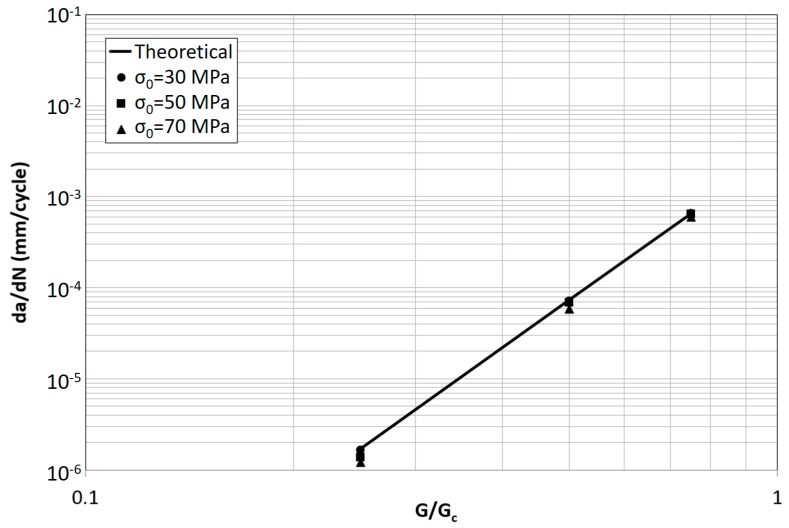
FCG rate as a function of G/G_c_ for different values of σ_0_ (K_0_ = 10^5^ MPa/mm).

Concerning the effect of σ_0_, the results are summarized in Figure 12 for K_0_ = 10^5^ MPa/mm. Also in this case the FCG rates respect the theoretical trend with a limited underestimation at the lowest value of G/G_c_.

Globally, it can be said that the modification proposed in Section 4.1 yields good results over a broad range of cohesive initial stiffness (K_0_) and cohesive strength (σ_0_) for a fixed value of the quasi-static critical value of strain energy release rate.

## 5. Conclusions

The model developed in [27], has been reviewed critically concerning damage evolution and implementation strategy. A modification to improve the accuracy of the model in yielding the theoretical FCG rate has been proposed and the response has been assessed by performing simulations varying the following model parameters:

- Initial stiffness K_0_
- Cohesive strength σ_0_

The results showed that the modified model is robust with respect to changes of two orders of magnitude in initial stiffness. The influence of cohesive strength σ_0_ was tested in the case of an intermediate value of stiffness K_0_ = 10^5^ MPa/mm and a limited difference between theoretical and numerically simulated FCG rate was found also in this case, for changes of a factor of two in σ_0_.

## Figures and Tables

**Table 1:** Cohesive zone model (CZM) and fatigue crack growth (FCG) parameters that are not varied in the simulations, from 21.

Parameter	Value
G_c_ [N/mm]	0.26
∆G_th_ [N/mm]	0.06
σ_0_ [MPa]	30
δ_c_[mm]	0.0173
B [mm/cycle × (N/mm)^−d^]	4.443
d	5.4

**Table 2:** CZM parameters that are varied in the simulations.

Parameter	Value		
K_0_ [MPa/mm]	10^4^	10^5^	10^6^
	30	30	30
σ_0_ [MPa]		50	
		70	

## Nomenclature

A	crack area
A_d_	damaged area produced by voids or cracks within a Representative Interface Element (RIE)
A_d_*^j^	damaged area related to ∆D* for the j-th increment
A_e_	area of a Representative Interface Element (RIE)
A_e,i_	effective area of the i-th elements
A_eff_	undamaged length of the crack tip element when a new loading is applied
A_fat_	portion of A_CZ_ where damage develops subcritically (due to fatigue)
A_CZ_	total section area of cohesive elements where D > 0 (process zone)
A_CZ,s_	quasi-static cohesive zone length
A_cz_^j^	quasi-static cohesive zone length for the j-th increment
B	fatigue crack growth rate coefficient
D	damage variable representing the loss of stiffness
D_f_	fatigue damage
D^j,i^	damage for the i-th integration point, j-th increment
D_s_	quasi static damage
$\overset{-}{D}$	damage variable representing the ratio of energy dissipated during the damage process
Ḋ	overall damage rate
D*^j,i^	corrected damage related to the explicit damage update procedure for the i-th integration point, j-th increment
E	adherends elastic modulus
G	strain energy release rate
G^j^	strain energy release rate for the j-th increment
G_c_	quasi-static critical value of strain energy release rate
G_th_	threshold strain energy release rate
G_max_	maximum value of the strain energy release rate in a cycle
I	second moment of area of the beam section
K	damaged cohesive stiffness
K_0_	initial (undamaged) cohesive stiffness
N	number of cycles
N^j^	number of cycles at the j-th increment
R	load ratio of the fatigue cycle (minimum load of the cycle/maximum load of the cycle)
d	fatigue crack growth rate exponent
l_CZ_	length of cohesive zone ahead of the crack tip
n_CZ_	number of IPs lying within A_CZ_
r_w_	relative weight of the integration point ([34,35])
∆D^j,i^	damage variation for the i-th integration point, j-th increment
∆D_max_	maximum value of fatigue damage increment set by the user
∆D*^j,i^	fatigue damage increment related to the explicit damage update procedure for the i-th integration point, j-th increment
∆G	cyclic strain energy release range
∆G^j^	cyclic strain energy release range of the j-th increment
∆G_th_	cyclic threshold strain energy release rate
∆N	increment in the number of cycles related to ∆D
∆N^j,i^	increment in the number of cycles for the i-th integration point, j-th increment, related to ∆D^j,i^
∆N^j^_min_	minimum of the for the ∆N^j,i^ j-th increment
∆N*^j^	variation of the number of cycles related to the explicit damage update procedure for the j-th increment
∆N*	increment in the number of cycles related to ∆D*
Ξ	energy dissipated during the damage process
δ	opening
δ^j,i^	opening for the i-th integration point, j-th increment
δ_0_	threshold opening for quasi-static crack growth
δ_0_^j,i^	threshold opening for the i-th integration point, j-th increment
δ_c_	critical (maximum) opening for quasi-static crack growth
δ_th_	threshold opening for fatigue crack growth
σ	stress at the integration point
σ^j,i^	stress for the i-th integration point, j-th increment
σ_0_	threshold stress for quasi-static crack growth
